# Tetrathiomolybdate Treatment Attenuates Bleomycin-Induced Angiogenesis and Lung Pathology in a Sheep Model of Pulmonary Fibrosis

**DOI:** 10.3389/fphar.2021.700902

**Published:** 2021-10-22

**Authors:** Habtamu B. Derseh, Kopiyawaththage U. E. Perera, Sasika N. Vithana Dewage, Andrew Stent, Emmanuel Koumoundouros, Louise Organ, Charles N. Pagel, Ken J. Snibson

**Affiliations:** ^1^ Faculty of Veterinary and Agricultural Sciences, University of Melbourne, Parkville, VIC, Australia; ^2^ Faculty of Veterinary and Agricultural Sciences, University of Melbourne, Werribee, VIC, Australia; ^3^ Department of Biomedical Engineering, Melbourne School of Engineering, University of Melbourne, Parkville, VIC, Australia; ^4^ Division of Respiratory Medicine, University of Nottingham, Nottingham, United Kingdom

**Keywords:** Angiogenesis, Bleomycin, Copper, Large animal model, Pulmonary fibrosis, Tetrathiomolybdate

## Abstract

Idiopathic pulmonary fibrosis (IPF) is a progressive chronic lung disease characterized by excessive extracellular matrix (ECM) deposition in the parenchyma of the lung. Accompanying the fibrotic remodeling, dysregulated angiogenesis has been observed and implicated in the development and progression of pulmonary fibrosis. Copper is known to be required for key processes involved in fibrosis and angiogenesis. We therefore hypothesized that lowering bioavailable serum copper with tetrathiomolybdate could be of therapeutic value for treating pulmonary fibrosis. This study aimed to investigate the effect of tetrathiomolybdate on angiogenesis and fibrosis induced in sheep lung segments infused with bleomycin. Twenty sheep received two fortnightly infusions of either bleomycin (3U), or saline (control) into two spatially separate lung segments. A week after the final bleomycin/saline infusions, sheep were randomly assigned into two groups (*n* = 10 per group) and received twice-weekly intravenous administrations of either 50 mg tetrathiomolybdate, or sterile saline (vehicle control), for 6 weeks. Vascular density, expressed as the percentage of capillary area to the total area of parenchyma, was determined in lung tissue sections immuno-stained with antibodies against CD34 and collagen type IV. The degree of fibrosis was assessed by histopathology scoring of H&E stained sections and collagen content using Masson’s trichrome staining. Lung compliance was measured via a wedged bronchoscope procedure prior to and 7 weeks following final bleomycin infusion. In this large animal model, we show that copper lowering by tetrathiomolybdate chelation attenuates both bleomycin-induced angiogenesis and pulmonary fibrosis. Moreover, tetrathiomolybdate treatment downregulates vascular endothelial growth factor (VEGF) expression, and improved lung function in bleomycin-induced pulmonary fibrosis. Tetrathiomolybdate also suppressed the accumulation of inflammatory cells in bronchoalveolar lavage fluid 2 weeks after bleomycin injury. The molecular mechanism(s) underpinning copper modulation of fibrotic pathways is an important area for future investigation, and it represents a potential therapeutic target for pulmonary fibrosis.

## Introduction

Idiopathic pulmonary fibrosis (IPF) is a progressive chronic lung disease of unknown cause. It is a devastating illness with a median survival time after diagnosis of 2–4 years (Ley et al., 2011). Although two disease retarding drugs have been approved for IPF in 2014, there is no totally effective pharmacologic treatment currently available that cures the disease (King et al., 2014; Richeldi, 2014; Richeldi et al., 2014). IPF exhibits complex pathogenesis which involves abnormal wound healing process and extracellular matrix (ECM) remodeling in the parenchyma of the lung. Healthy lung tissue is replaced by ECM deposition and both alveolar architecture and pulmonary microvasculature are destroyed leading to loss of lung compliance, disruption of gas exchange, respiratory failure and ultimately death (Richeldi et al., 2017). Accompanying the fibrotic remodeling, aberrant vascular remodeling associated with angiogenesis has been observed in both tissue specimens from IPF patients and animal models of pulmonary fibrosis (Peão et al., 1994; Ebina, 2017).

The presence of vascular remodeling in IPF was first described by Turner-Warwick, who demonstrated that formation of new blood vessels and anastomoses between the systemic and pulmonary microvasculature in lung specimens from patients with pulmonary fibrosis (Turner-Warwick, 1963). Subsequent studies have also observed vascular remodeling in pulmonary fibrosis, and thus give rise to the idea that neoformation of blood vessels may be important for the development of pulmonary fibrosis (Peão et al., 1994; Renzoni et al., 2003). An imbalance between angiogenic and angiostatic cytokines favoring net angiogenesis has been demonstrated in lung tissues of IPF patients, and also in bleomycin mouse models of pulmonary fibrosis (Keane et al., 1997; Keane et al., 1999a; Keane et al., 1999b; Keane et al., 2001). Neutralization of angiogenic factors has been shown to reduce bleomycin-induced pulmonary fibrosis through a reduction in angiogenesis (Keane et al., 1999a). Therefore, targeting these cytokines might be helpful in pulmonary fibrosis.

Copper is an essential trace element for all forms of life and performs various biological functions. Copper-mediated mechanisms are implicated in the expression, activation, secretion, and binding of key angiogenic cytokines such as vascular endothelial growth factor (VEGF), fibroblast growth factor 2 (FGF2) and platelet-derived endothelial cell growth factor (PD-ECGF) (Brewer, 2001; Brewer, 2005a; Goodman et al., 2005; Gupte and Mumper, 2009). Similarly, cytokines involved in inflammation and fibrosis, such as tumor necrosis factor (TNF)-α and transforming growth factor (TGF)-β1, can be inhibited by lowering copper concentration (Brewer, 2003; Brewer et al., 2004; Brewer, 2005b). When copper levels are reduced to 50–70% of baseline, cytokine signaling is inhibited but house-keeping cellular processes are unimpaired (e.g., copper-dependent enzymes such as lysyl oxidase, cytochrome oxidase etc will not be affected at these lower copper levels) (Brewer, 2001; Brewer, 2005a; Goodman et al., 2005), suggesting copper-lowering strategies may provide a potential therapeutic approach for IPF.

Tetrathiomolybdate (TM) is a highly specific and potent copper chelating drug that was initially developed as a therapeutic agent to treat Wilson’s disease, a copper storage genetic disorder, characterized by excessive copper accumulation in liver and brain (Brewer et al., 1991; Brewer et al., 1994). The mechanism of copper reduction of TM involves chelation of free copper with itself and albumin/protein forming a tripartite complex which is unavailable for cellular uptake. TM has been demonstrated to safely reduce the bioavailability of copper within 2–4 weeks of treatment in mice, rats, sheep and humans (Gooneratne et al., 1981; MIlls et al., 1981; Brewer et al., 2000; Brewer et al., 2003). The expression of several pro-angiogenic, pro-inflammatory and pro-fibrotic cytokines, including IL-1, IL-6, IL-8, basic fibroblast growth factor (bFGF), and VEGF, are inhibited by copper-lowering therapy with TM (Pan et al., 2002; Pan et al., 2003). This occurs through multiple mechanisms including suppression of the NF-κB signaling pathway (Pan et al., 2002; Pan et al., 2003).

In our previous study, we have demonstrated the presence of microvascular remodeling and elevated VEGF expression in the sheep model of bleomycin-induced pulmonary fibrosis (Derseh et al., 2019). In the current study, we hypothesized that lowering bioavailable serum copper with TM could be of therapeutic value for pulmonary fibrosis by inhibiting microvascular remodeling and the expression of pro-angiogenic mediators. The overall objective of this study is to analyze the effect of TM on angiogenesis and fibrosis development in sheep lung segments infused with bleomycin. This study examines both the pathology and lung functional changes resulting from TM treatment.

## Materials and Methods

### Sheep Model of Bleomycin-Induced Pulmonary Fibrosis

**FIGURE 1:**
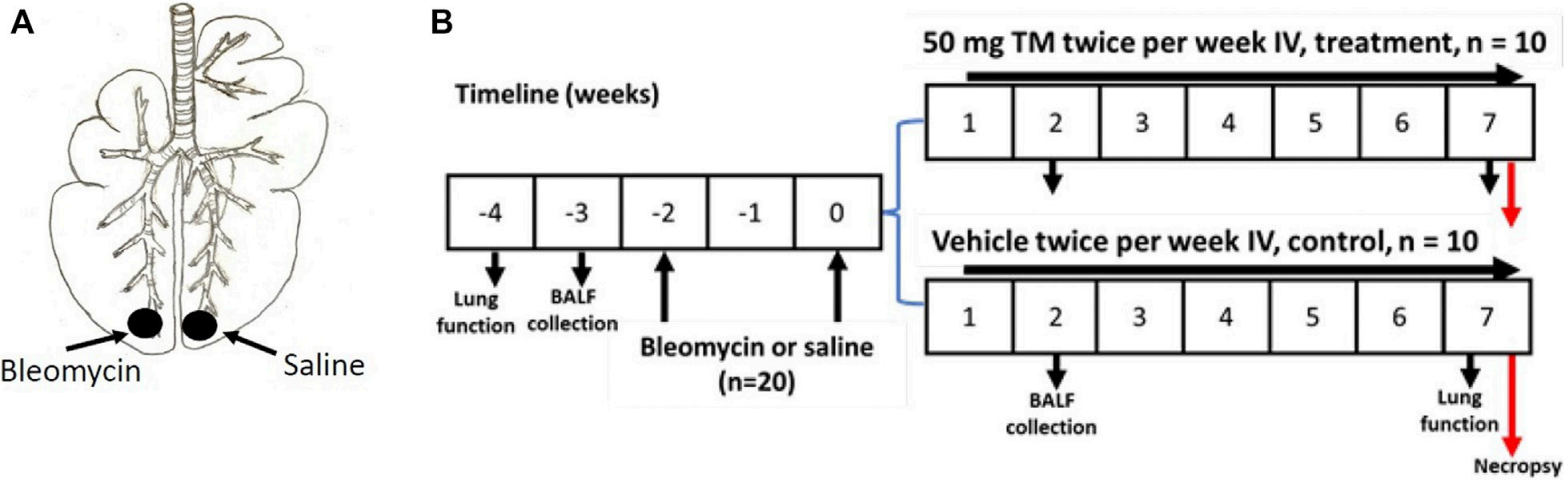
Induction of pulmonary fibrosis and administration of TM in sheep. **(A)** Schematic illustration showing the bleomycin and saline infusions into the caudal lung segments of animals. In each animal, while one caudal lung segment was infused with bleomycin, the contralateral segment was infused with sterile saline to act as an internal control. **(B)** Diagram displaying the timeline for IV treatment of TM, or vehicle (sterile saline), lung function analysis, collection of BAL fluid, and euthanasia of experimental animals.

Twenty female Merino sheep were used for the current study. The sheep were approximately aged 1 year, and all experiments conducted on sheep were approved by the Animal Ethics Committee of the University of Melbourne (Parkville, VIC, Australia). Two doses of bleomycin sulfate (3U bleomycin in 5 ml sterile saline (Bleomycin; Hospira, Melbourne, VIC, Australia), 2 weeks apart, were infused into the caudal segments of lungs of all sheep to induce fibrosis as previously described (Organ et al., 2015a; Organ et al., 2015b; Organ et al., 2017); (Figure 1A). Sheep were euthanized 7 weeks after bleomycin injury by an IV overdose of barbiturate (Lethabarb, Virbac Animal Health, Australia) and the tissue was collected and stored in the freezer at −80°C until required for further analysis. The timeline of the experiment is shown in Figure 1B.

### TM Treatment of Sheep

One week after the second bleomycin/saline infusion, sheep were randomly assigned to either vehicle- or tetrathiomolybdate-treated groups (*n* = 10 each group). Treatment consisted of either twice weekly IV injection of tetrathiomolybdate (50 mg TM; Sigma Aldrich) in vehicle solution (sterile saline), or vehicle alone (sterile saline) for 6 weeks, depending on the treatment group (Figure 1B). TM solution was prepared by dissolving TM crystals in sterile saline as a 50 mg/ ml solution and stored as 5- or 10-ml portions in stoppered sterile vials at -20°C. On the day of injection, the solutions were thawed at room temperature, centrifuged and the supernatant fraction was used for intravenous administration as previously described (Gooneratne et al., 1981).

### Copper Status

Copper status was assessed by analyzing serum ceruloplasmin, a copper-containing ferroxidase, based on its oxidase activity in serum from the sheep jugular vein. In the presence of TM therapy, serum or tissue concentration of copper is not useful since a tripartite complex of tetrathiomolybdate, copper, and albumin is still measurable but not available for cellular uptake. Serum ceruloplasmin level has been used previously as a surrogate marker of bioavailable copper level because the liver releases this enzyme into the bloodstream at a rate that is dependent on the availability of copper (Brewer et al., 2000; Lowndes et al., 2009).

### Analysis of Segmental Lung Function

Segmental lung compliance analysis was performed in separate lung segments at baseline and 7 weeks after bleomycin infusion using a wedged-bronchoscope procedure in alert consciously breathing sheep as described previously (Organ et al., 2015a). In short, the resistance of the bronchoscope to the set flow was first determined, then the fiber optic bronchoscope was wedged into an airway in the caudal sheep lung segment. A constant flow of 6 ml/ s 5% CO_2_ in air was passed through the bronchoscope working channel and a steady-state pressure was allowed to build up. Five seconds after a steady-state pressure reached, the airflow was interrupted by shutting it off from the supply. Segmental compliance was determined from the flow-pressure decay curve produced from this procedure, as described previously (Cetti et al., 2006; Tsai et al., 2007; Organ et al., 2015a). Segmental compliance values were corrected during calculation by reducing the resistance of airflow through the bronchoscope working channel.

### Collection of Bronchoalveolar Lavage

Bronchoalveolar (BAL) fluid was collected from all animals prior to commencement of bleomycin infusion and then again 2 weeks after the second bleomycin dose as outlined in Figure 1. BAL fluid was collected by advancing a fiber-optic bronchoscope into the caudal lung segments of interest and by instilling and withdrawal of 10 ml of sterile saline solution. The lavage fluid was then placed in test tube and put on ice immediately. The lavage fluid samples were centrifuged at 1,000 rpm for 7 min to separate cells. Total and differential leukocyte counts were then done according to the previously described method (Bischof et al., 2003; Organ et al., 2015a). Total leukocyte number in the BAL fluid was measured by an automated cell counter (ZTM Series Coulter Counter VR Cell and Particle Counter, Beckman Coulter Inc. USA). Cytospots were prepared from BAL fluid using cytospin (Shandon Cytospin; Thermo Fisher Scientific) and then stained with Haemkwik (HD Scientific, Australia) for differential leukocyte counts.

### Necropsy and Tissue Sampling

Sheep were euthanized by IV overdose of barbiturate and then lung tissues were dissected out. The targeted individual segments were cut and inflated with a 1:1 mixture of sterile PBS: optimal cutting temperature compound solution by pumping this solution through the bronchi. This technique maintains lung tissue in inflated condition before fixation for paraffin processing and prior to freezing for cryo-sectioning. Several 0.5 cm thick transverse lung tissue blocks were cut. Slices from the most caudal part of all differentially treated lung lobes were fixed in 4% neutral-buffered formalin and processed in paraffin for the assessment of histopathology. The rest of the lung tissue blocks were embedded in optimal cutting temperature compound and snap-frozen in liquid nitrogen. These lung samples were then kept at −80°C until required for further analysis.

### Immunohistochemistry

Immunohistochemistry was conducted on frozen sheep lung tissue sections using previously described methods (Organ et al., 2017). Primary antibodies and optimal dilutions used in the current study were: anti-human collagen type IV (mouse monoclonal, 1:50, Dako, Kingsgrove, Australia); anti-CD34 (rabbit monoclonal, 1:50, Abcam, Waterloo, Australia); anti-human VEGF (A-20) (rabbit polyclonal, 1:50, Abcam). Secondary antibodies and their dilutions were: anti-mouse rabbit IgG (HRP) (ab6728), 1:100 and anti-rabbit goat IgG/HRP, 1:100 (both from Abcam). Primary antibody was omitted for negative controls which were included during each immunohistochemistry procedure. Counterstaining was performed using Mayer’s Haematoxylin solution and 3,3′-diaminobenzidine was used to visualize antigen-antibody reaction.

### Quantitative Evaluation of Vascularity

Vascularity of the lung parenchyma was evaluated quantitatively in all differentially treated lung tissue sections immuno-stained with blood vessel markers, anti-CD34 and anti-collagen type IV antibodies. Ten images were captured at x400 magnification from fibroproliferative areas using a digital camera (Leica ICC50 W, Leica Microsystems, Wetzlar, Germany) linked to a light microscope (Leica DM 500, Leica Microsystems, Wetzlar, Germany) for each lung tissue section treated differentially. Bronchi and large blood vessels were excluded during image capturing. The analysis of all images was performed using Image-Pro^®^ Plus software (Version 6.3 for Windows, Media Cybernetics, Bethesda, Maryland, United States) by HBD in a blinded manner without knowing the treatment groups. Vascular density was represented by the percentage of the total area occupied by CD34 and collagen type IV positive endothelial cells per total area of parenchyma. The results of vascular density for each of the ten images were then averaged for each lung tissue section.

### Histopathology

The assessment of histopathological changes was performed on haematoxylin and eosin (H and E) stained sections, and changes to connective tissue content in the lung parenchyma was assessed using Masson’s trichrome stained sections as previously described (Organ et al., 2015a). Histopathology of lung parenchyma was examined by an experienced veterinary pathologist (AS) in a blinded manner on coded slides using a previously described semi-quantitative scoring system (Organ et al., 2015a). In short, the procedure for blinding included digitally scanning of lung tissue sections from all differentially treated lung segments, and then each digitally scanned image was given a random, non-descriptive, de-identifying code prior to assessment by a veterinary pathologist (AS). The treatment groups were only identified after all the assessments had been conducted and the data had been gathered. Ten random H and E-stained fields which lacked large bronchi and blood vessels were captured at 200x magnification for each differentially treated lung tissue section in all animals. The inflammation and fibrosis were scored separately, and these score values were added together to give an ‘overall pathology score’. Score grades were defined as: 0: absent, 1: <25%, 2: 25–50%, 3: >50% involvement. Results from all ten images were averaged to give a representative score for overall pathology for each differentially treated lung tissue section.

The collagen content in the lung parenchyma which indicates the degree of fibrosis was quantified using a previously described method (Izbicki et al., 2002). Briefly, slides stained with Masson’s trichrome were digitally scanned using a Mirax slide scanner (Carl Zeiss Micro-Imaging, Jena, Germany). Ten random fields without large bronchi and blood vessels were captured at x200 magnification. In each image, blue-stained tissue area (collagen) was measured within the parenchyma using Image-Pro Plus software. The percentage of blue-stained areas per total field area for each of the ten images was then averaged for each differentially treated lung tissue section.

Lung parenchymal tissue percentage was analyzed in a blinded manner by Image-Pro Plus software using custom-designed test grids. In short, images of H and E-stained lung tissue sections from all differentially treated lung segments were exported into Image-Pro Plus and analysis was performed by superimposing Image-Pro Plus generated custom-designed test grids over the images. Point-counting method was used to determine tissue percentage within the lung parenchyma as described previously (Maritz et al., 2008; Atik et al., 2012). For each differentially treated lung tissue section, ten images captured at x200 magnification were analyzed and the results were then averaged for each section.

### Statistical Analysis

Statistical analysis was done using GraphPad Prism for Windows Version 5.01 (GraphPad Software Inc., La Jolla, CA, United States). Each parameter was evaluated for Gaussian distribution using the Pearson omnibus and D’Agostino normality test. For parametric data, paired two-tailed *t*-tests were performed to compare between bleomycin-infused and saline-infused control segments in the same sheep whereas the Wilcoxon matched-pairs signed rank test was used for data that did not meet assumptions for Gaussian distribution. Differences between the drug (TM) treated sheep and vehicle (sterile saline) treated sheep were analyzed using an unpaired *t*-test for parametric data and a Mann-Whitney test for nonparametric data. A *p*-value less than 0.05 was defined as a significant difference. All values are reported as means ± SEM.

## Results

### Tetrathiomolybdate Treatment Inhibited Bleomycin-Induced Angiogenesis

**FIGURE 2:**
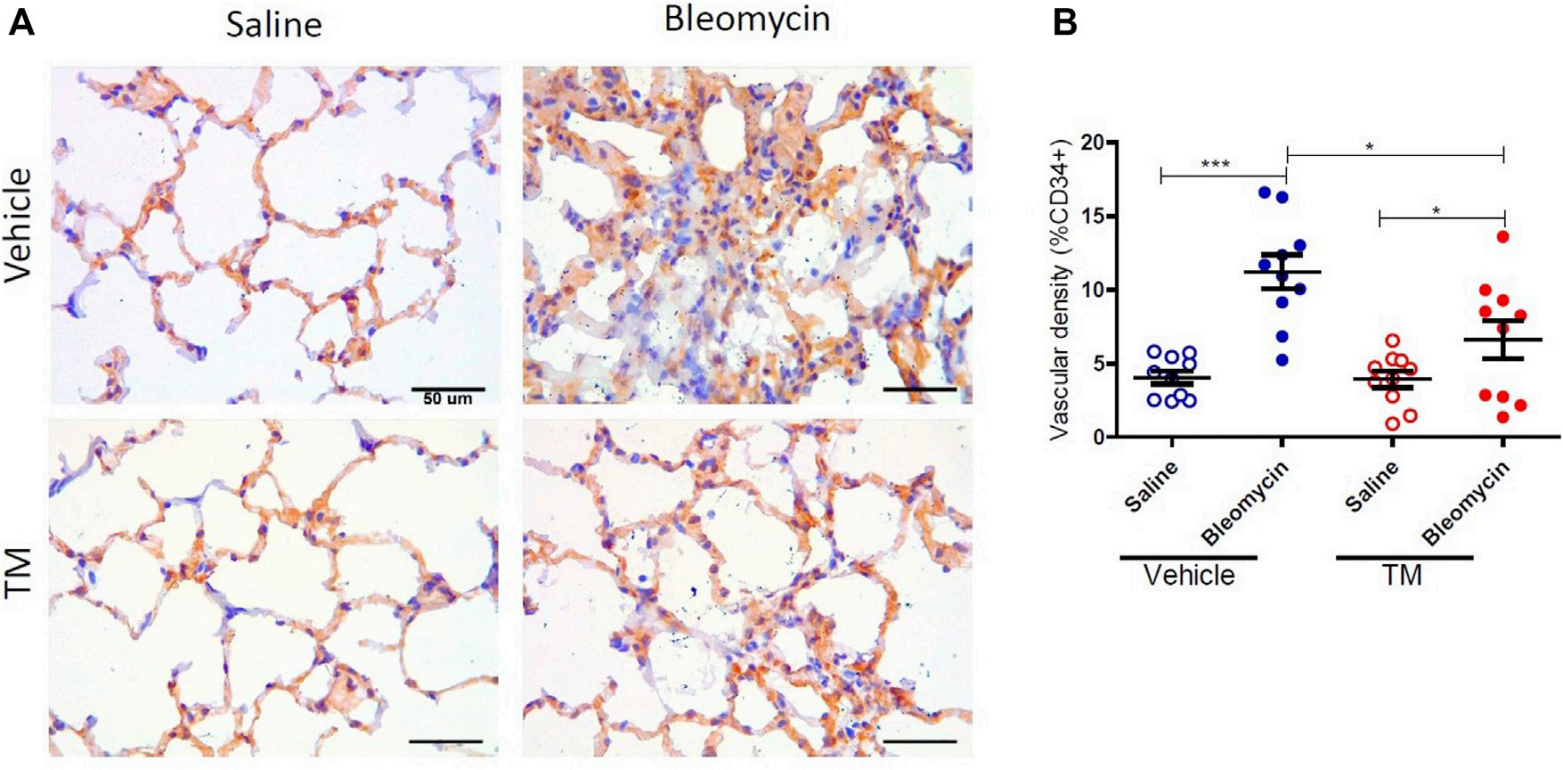
Effects of tetrathiomolybdate on bleomycin-induced angiogenesis of CD34 positive capillaries. **(A)** Representative photomicrographs showing anti-CD34 immuno-stained lung tissue sections of saline and bleomycin-infused lung segments from either vehicle (sterile saline) or tetrathiomolybdate treated sheep. In each animal, one lung segment was infused with saline (left-side images) whereas the contralateral segment was infused with bleomycin (right-side images). **(B)** Shows the vascular density data of differentially treated lung tissue segments of either vehicle or tetrathiomolybdate treated animals. Significance was analyzed using a paired *t*-test between saline-*vs*. bleomycin-infused segments from the same animals and an unpaired *t*-test between segments from different animals (vehicle vs TM treated animals), *p < 0.05, ***p < 0.001, (*n* = 10 Sheep). Data represent mean ( ± SEM). Scale bars = 50 µm.

**FIGURE 3:**
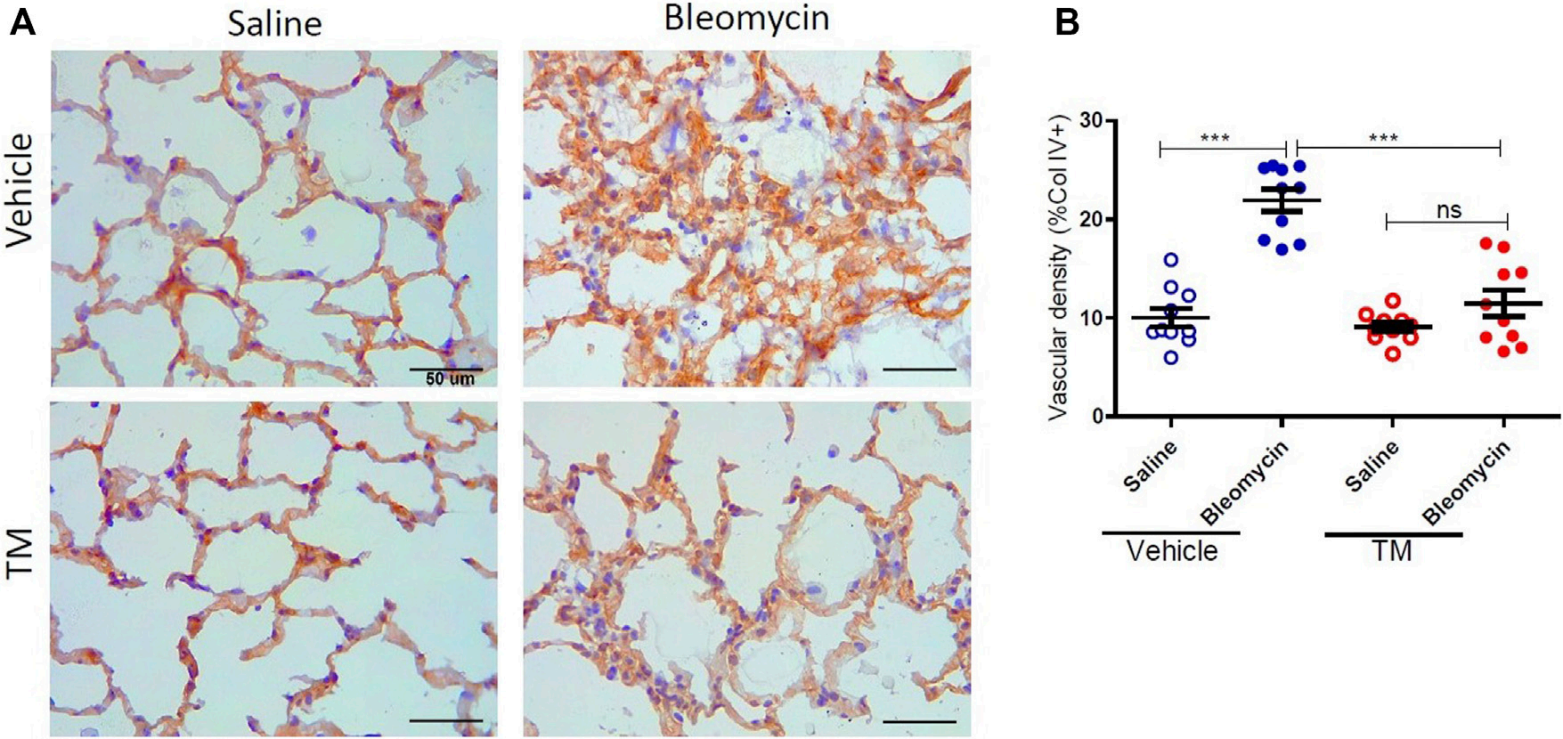
Effects of tetrathiomolybdate on bleomycin-induced angiogenesis of collagen type IV positive capillaries. **(A)** Representative photomicrographs showing anti-type IV collagen immuno-stained lung tissue sections of saline and bleomycin-infused lung segments from either vehicle (sterile saline) or tetrathiomolybdate treated sheep. In each animal, one lung segment was infused with saline (left-side images) whereas the contralateral segment was infused with bleomycin (right-side images). **(B)** Shows the vascular density data of differentially treated lung tissue segments of either vehicle or tetrathiomolybdate treated animals. Significance was analyzed using a paired *t*-test between saline-vs bleomycin-infused segments from the same animals and an unpaired *t*-test between segments from different animals (vehicle *vs*. TM treated animals), ***p < 0.001, ns-not significant, (*n* = 10 Sheep). Data represent mean ( ± SEM). Scale bars = 50 µm.

Serum ceruloplasmin was assayed as a surrogate marker of copper status. Ceruloplasmin concentration was significantly reduced following TM treatment and at week seven, ceruloplasmin concentration was 73% of baseline values as shown in the supplementary information (Supplementary Figure S1). Angiogenesis was assessed using two blood vessel markers, namely, CD34 and collagen type IV, as previously described (Derseh et al., 2019). When assessed by CD34, vehicle-treated animals displayed significantly higher microvascular density in bleomycin-infused segments compared to saline-infused control segments (4.06 ± 0.45 saline-vehicle *vs*. 11.23 ± 1.16 bleomycin-vehicle), *p* = 0.0001 (Figure 2A + B). In TM-treated animals, there was also higher microvascular density in bleomycin-infused lobes compared to saline-infused segments (6.62 ± 1.3 bleomycin-TM *vs*. 3.93 ± 0.56 saline-TM), *p* = 0.035 (Figure 2B). However, in bleomycin injured segments, TM-treated animals had significantly less microvascular density compared to vehicle only treated animals (11.23 ± 1.16 bleomycin-vehicle *vs*. 6.62 ± 1.3 bleomycin-TM), *p* = 0.0162 (Figure 2B). Similarly, in vehicle-control sheep, there was significantly increased density of collagen type IV-positive capillaries in the parenchyma of bleomycin-infused segments compared to saline-infused control segments indicating neovascularization of collagen type IV-positive capillaries is also induced by bleomycin-infusion (10.02 ± 0.93% saline *vs*. 21.94 ± 1.12% bleomycin, *p* < 0.0001) (Figure 3A +B). This augmentation of collagen type IV-positive blood vessel density was significantly reduced by TM treatment (21.94 ± 1.12% bleomycin-vehicle *vs*. 11.45 ± 1.32% bleomycin-TM, *p* < 0.0001) (Figure 3B).

### Tetrathiomolybdate Treatment Downregulates Bleomycin-Induced VEGF Expression

**FIGURE 4:**
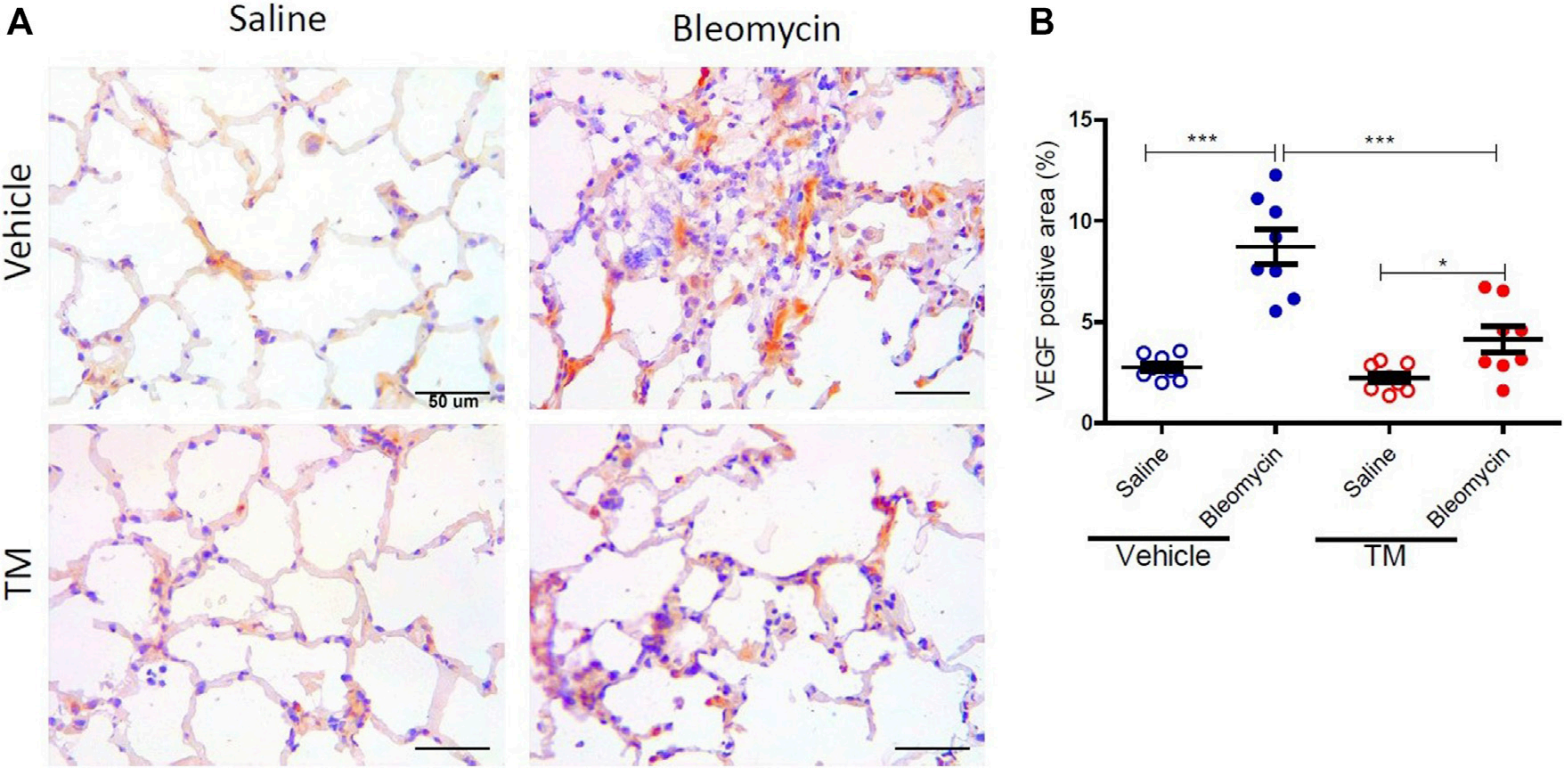
Tetrathiomolybdate downregulates bleomycin-induced VEGF expression. **(A)** Representative photomicrographs showing anti-VEGFA immuno-stained lung tissue sections of saline and bleomycin-infused lung segments from either vehicle (sterile saline) or tetrathiomolybdate treated sheep. In each animal, one lung segment was infused with saline (left-side images) whereas the contralateral segment was infused with bleomycin (right-side images). **(B)** Shows the data for the percentage VEGF-stained area per total area of the lung parenchyma in the field of view for the different treatment groups. Significance was analyzed using a paired *t*-test between saline-*vs*. bleomycin-infused segments from the same animals and an unpaired *t*-test between segments from different animals (vehicle *vs*. TM treated animals), *n* = 10 sheep, *p < 0.05, ***p < 0.001. Scale bars = 50 µm.

VEGF expression as indicated by immunohistology was visually more abundant in bleomycin-infused lung segments compared to saline-infused control segments in vehicle-treated animals (Figure 4A). The expression of VEGF in bleomycin-infused segments of TM-treated sheep was reduced to near saline control levels (Figure 4A). Analysis of the percentage of the VEGF-positive area per total field of lung parenchyma, revealed that there was significantly higher VEGF expression in bleomycin-infused segments than saline-infused segments in vehicle only treated animals (saline-vehicle 2.76 ± 0.21 *vs*. bleomycin-vehicle 8.73 ± 0.86, *p* = 0.0002) (Figure 4B). In TM-treated animals, there was also higher VEGF expression in bleomycin-infused than saline-infused segments (saline-TM 2.22 ± 0.24 *vs*. bleomycin-TM 4.13 ± 0.64, *p* = 0.0370) (Figure 3B). A comparison between tetrathiomolybdate and vehicle treatment groups showed that there was a two-fold reduction in VEGF expression in bleomycin-infused lung segments (bleomycin-vehicle 8.73 ± 0.86 *vs*. bleomycin-TM 4.13 ± 0.64, *p* = 0.0007) (Figure 4B).

### Tetrathiomolybdate Treatment Reduces Bleomycin-Induced Inflammatory Cell Accumulation in BAL Fluid

**FIGURE 5:**
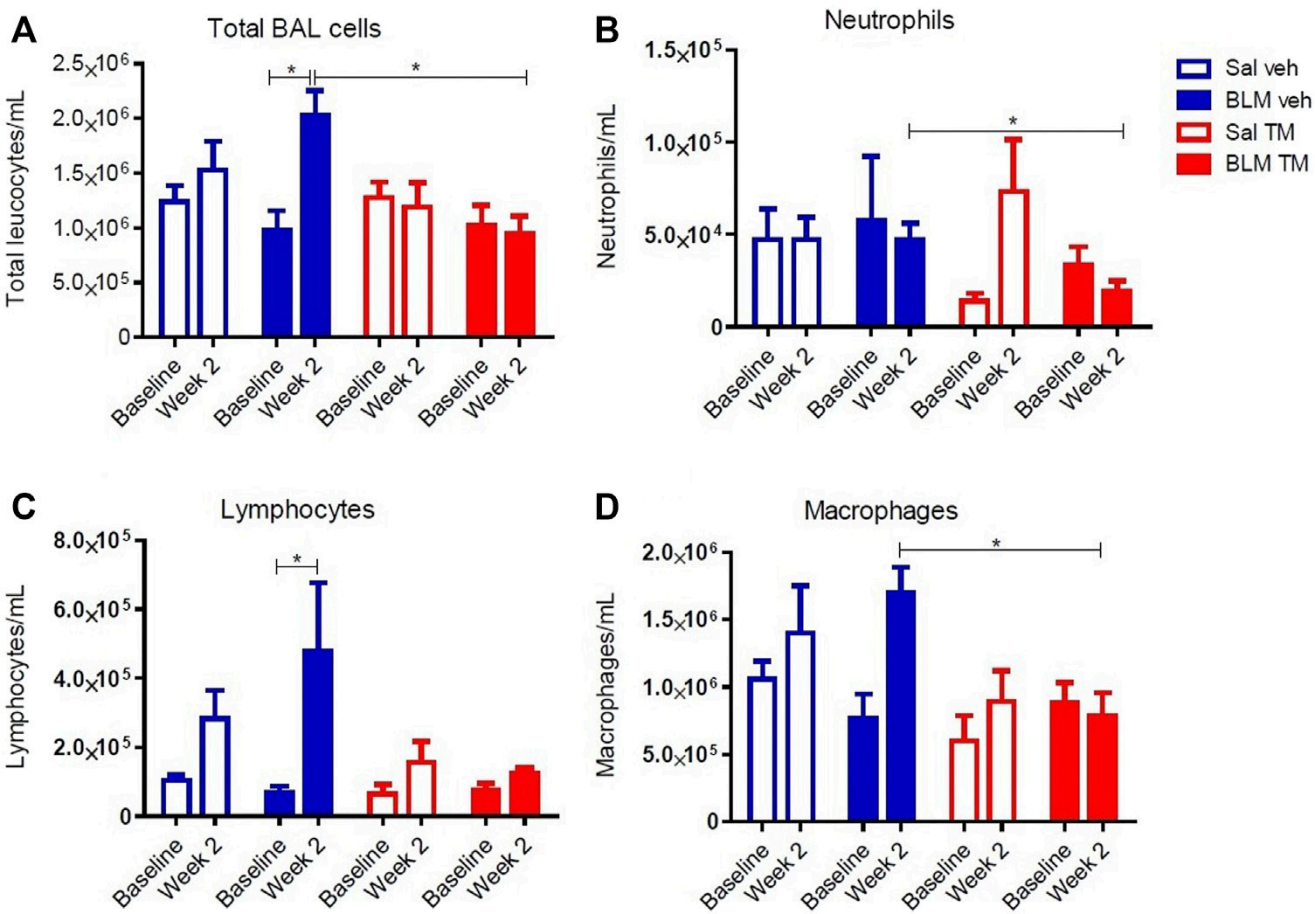
Total and differential leukocyte counts in BAL fluid. Total and differential leukocyte counts were performed on BAL cells collected from saline- and bleomycin-infused segments in sheep both prior to, and 2 weeks following the final infusion. **(A)** Total BAL cells, **(B)** Neutrophils, **(C)** Lymphocytes, and **(D)** Macrophages. Significance was determined using a paired *t*-test between segments from the same treatment group and an unpaired *t*-test between segments from different groups, *p < 0.05. Values are the mean ( ± SEM), *n* = 10 sheep.

BAL fluid was collected from differentially treated lung segments at baseline before the commencement of any treatment, and at 2 weeks following second bleomycin- or saline infusion (7 days into the TM/vehicle treatment regime). At 2 weeks after the final bleomycin infusions, there was a significant increase in the total numbers of leucocytes compared to pre-treatment values in the bleomycin-infused segments of vehicle only treated animals. TM treatment reduced inflammatory cell accumulation in BAL fluid (total leukocytes per mL of BAL: BLM-vehicle 2.02 × 10^6^ ± 233.96 × 10^3^ *vs*. BLM-TM 9.4 × 10^5^ ± 167.89 × 10^3^, *p* = 0.0205) (Figure 5A). Bleomycin induced a significant increase in lymphocytes after 2 weeks in the vehicle treated group, which was not observed in the TM-treated sheep (Figure 5C). Whilst not significant, bleomycin also increased macrophage counts after 2 weeks which was prevented in sheep treated with TM (Figure 5D).

### Tetrathiomolybdate Treatment Recovers the Loss of Pulmonary Function Associated With the Development of Bleomycin-Induced Pulmonary Fibrosis

**FIGURE 6:**
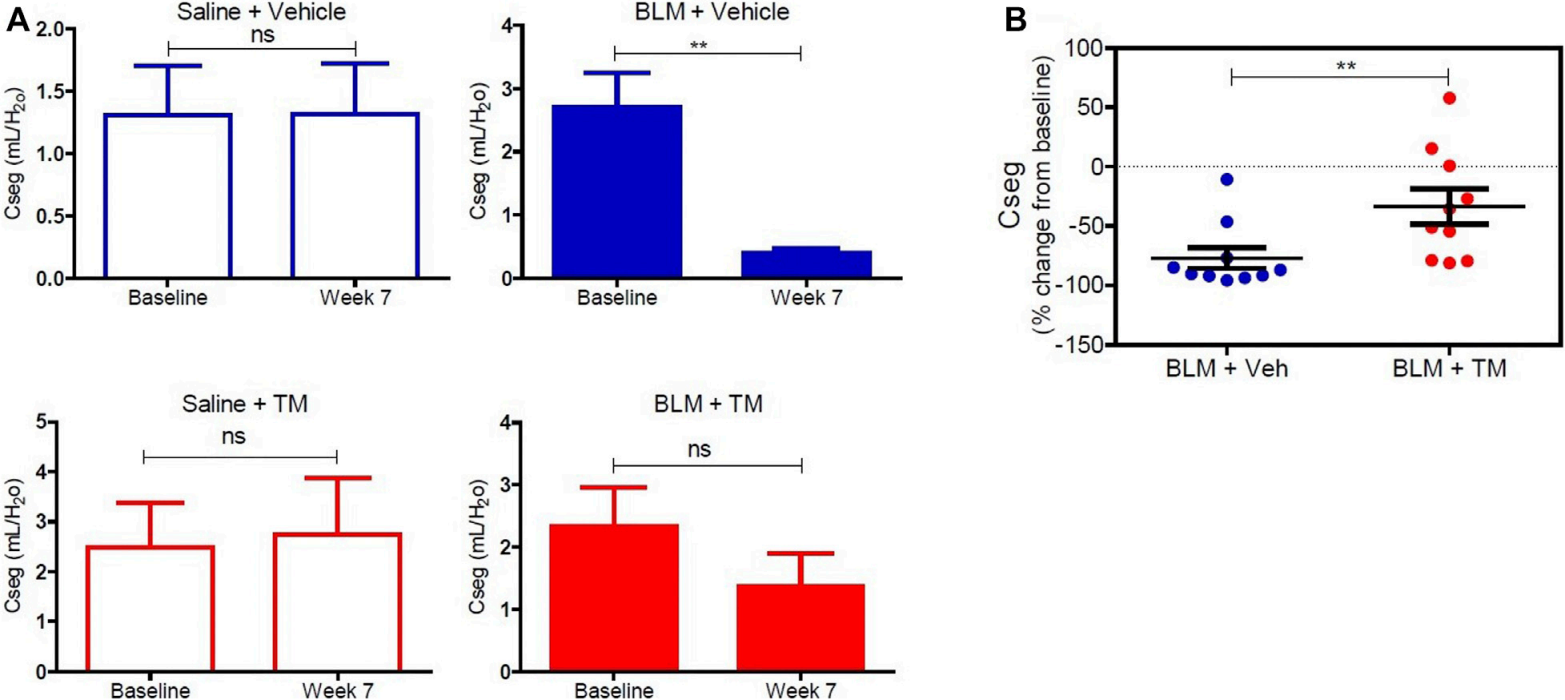
Lung compliance in differentially treated lung tissue sections. **(A)** Lung compliance at baseline and week seven post bleomycin injury in all differentially treated segments. Segments receiving saline and vehicle (saline + vehicle) control showed no significant difference in compliance. However, bleomycin-infused segments [with vehicle (sterile PBS), as a control for TM administration (bleomycin + vehicle)] showed a significant drop in compliance at 7 weeks from baseline. Saline segments that received TM (saline + TM) treatment showed no significant change. Bleomycin-infused segments that received TM (bleomycin + TM) showed comparable compliance at week seven and baseline. **(B)** A comparison of percent change from baseline at week seven in bleomycin injured segments of vehicle and TM treated animals. There is a significant increase in lung compliance in the sheep that received TM compared with the sheep treated with vehicle control only. Significance was determined using a paired *t*-test between lung segments from the same treatment group and an unpaired *t*-test between lung segments from different groups, **p < 0.01, ns-not significant. Data represent mean ( ± SEM).

Lung compliance was measured in each differentially treated lung segment before the commencement of any treatments, and at week seven after the second bleomycin instillation. There was no significant change in lung compliance at baseline and week seven in saline-infused segments in both vehicle- and TM-treated animals as shown in Figure 6A (saline-vehicle segment, baseline 1.31 ± 0.39 *vs*. 1.31 ± 0.41 ml/ cmH_2_0 weeks 7, *p* = 0.5566: saline-TM segment, baseline 2.47 ± 0.89 *vs*. 2.74 ± 1.13 ml/ cmH_2_0, week 7, *p* = 0.4922). A marked decline in segmental lung compliance as compared to baseline values was observed at week seven in the bleomycin-infused segments of vehicle control sheep (bleomycin-vehicle segment, baseline 2.71 ± 0.53 *vs*. 0.39 ± 0.08 ml/ cmH_2_0, week 7, *p* = 0.0027) (Figure 6A). In bleomycin segments of TM-treated sheep, lung compliance had increased near-baseline values by 7 weeks after final bleomycin-injury (bleomycin-TM segment, baseline 2.33 ± 0.63 *vs*. 1.36 ± 0.53 ml/ cmH_2_0, week 7, *p* = 0.1602) (Figure 6A). The percentage change in compliance from pre-bleomycin infusion values to 7 weeks after bleomycin injury was compared between the bleomycin-injured segments from both treatment groups. Figure 6B shows that sheep that received TM treatment had significantly higher lung compliance closer to pre-bleomycin injury baseline values, when compared to sheep treated with vehicle control only (Figure 6B).

### Effect of Tetrathiomolybdate Treatment on the Bleomycin-Induced Fibrotic Response

**FIGURE 7:**
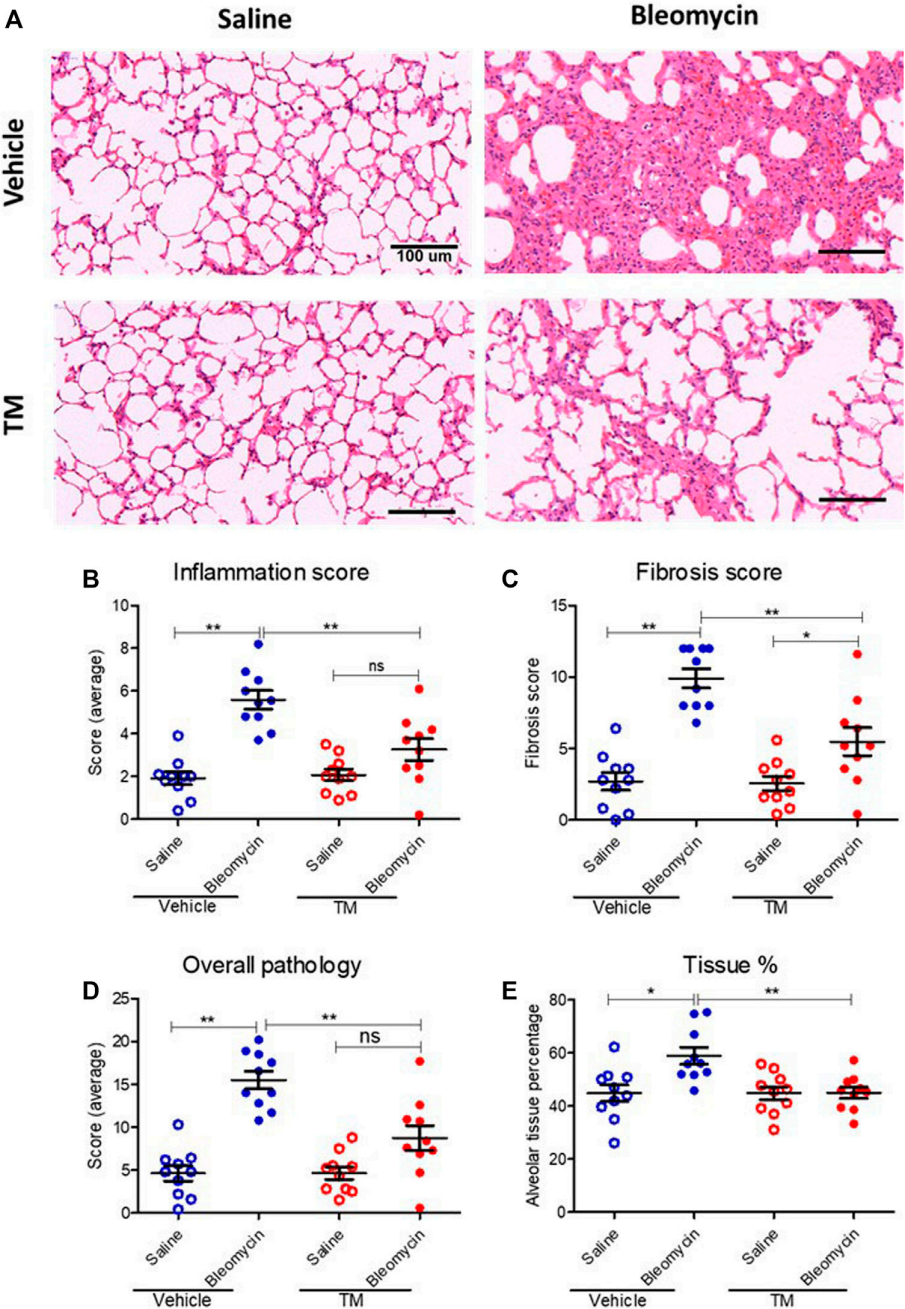
Histopathology changes in differentially treated lung tissue sections. **(A)** Representative photomicrographs of H and E-stained histological tissues in saline- or bleomycin-infused segments treated with either TM or vehicle. Pathology was quantified with a semiquantitative scoring system shown in **(B)** inflammation score only, **(C)** fibrosis score only, **(D)** overall pathology. **(E)** Graph showing tissue percentage in all differentially treated lung tissue sections. *p < 0.05, **p < 0.01. ns = not significant. Data represent mean (± SEM). Scale bars = 100 µm.

**FIGURE 8:**
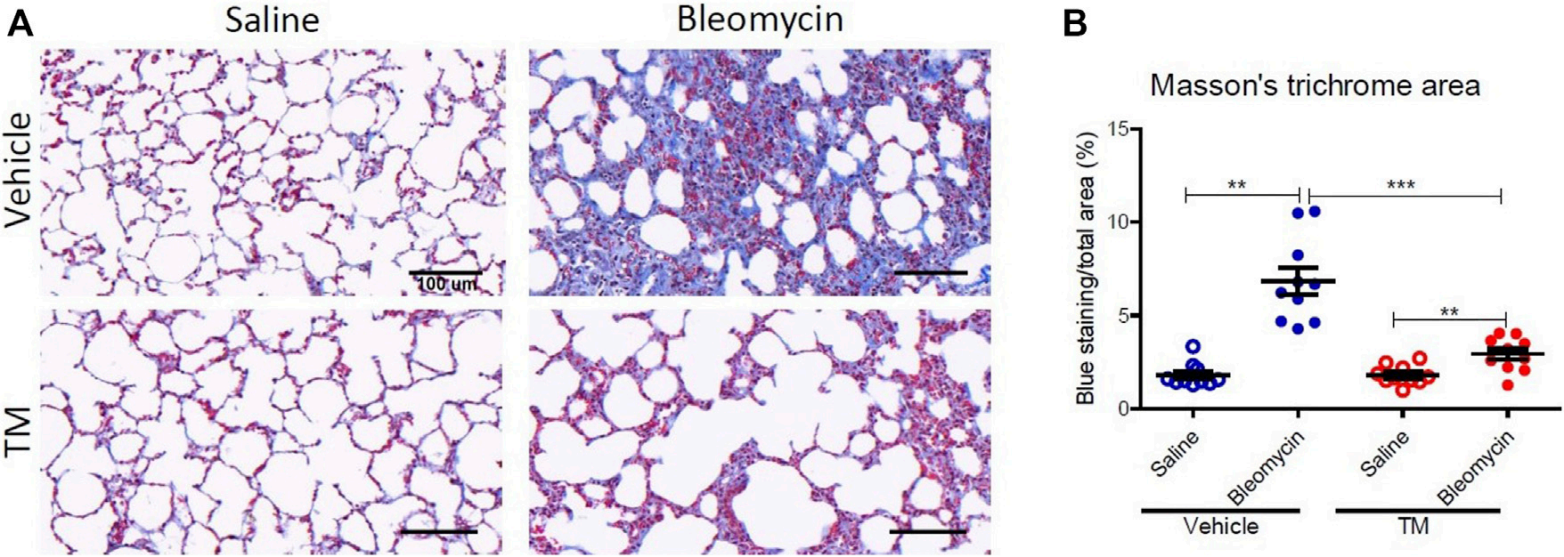
Collagen deposition in the parenchyma of differentially treated lung tissue sections. **(A)** Representative photomicrographs of Masson’s trichrome, blue-stained histological tissues in control and bleomycin challenged segments treated with either TM or vehicle. **(B)** Graphs showing fibrotic fraction (Masson’s trichrome blue staining area/total field area) in differentially treated lung tissue sections. Significance was determined using a paired *t*-test between lung segments from the same treatment group and an unpaired *t*-test between lung segments from different groups, **p < 0.01, ***p < 0.001. Data represent mean (± SEM). Scale bars = 100 µm.

**FIGURE 9:**
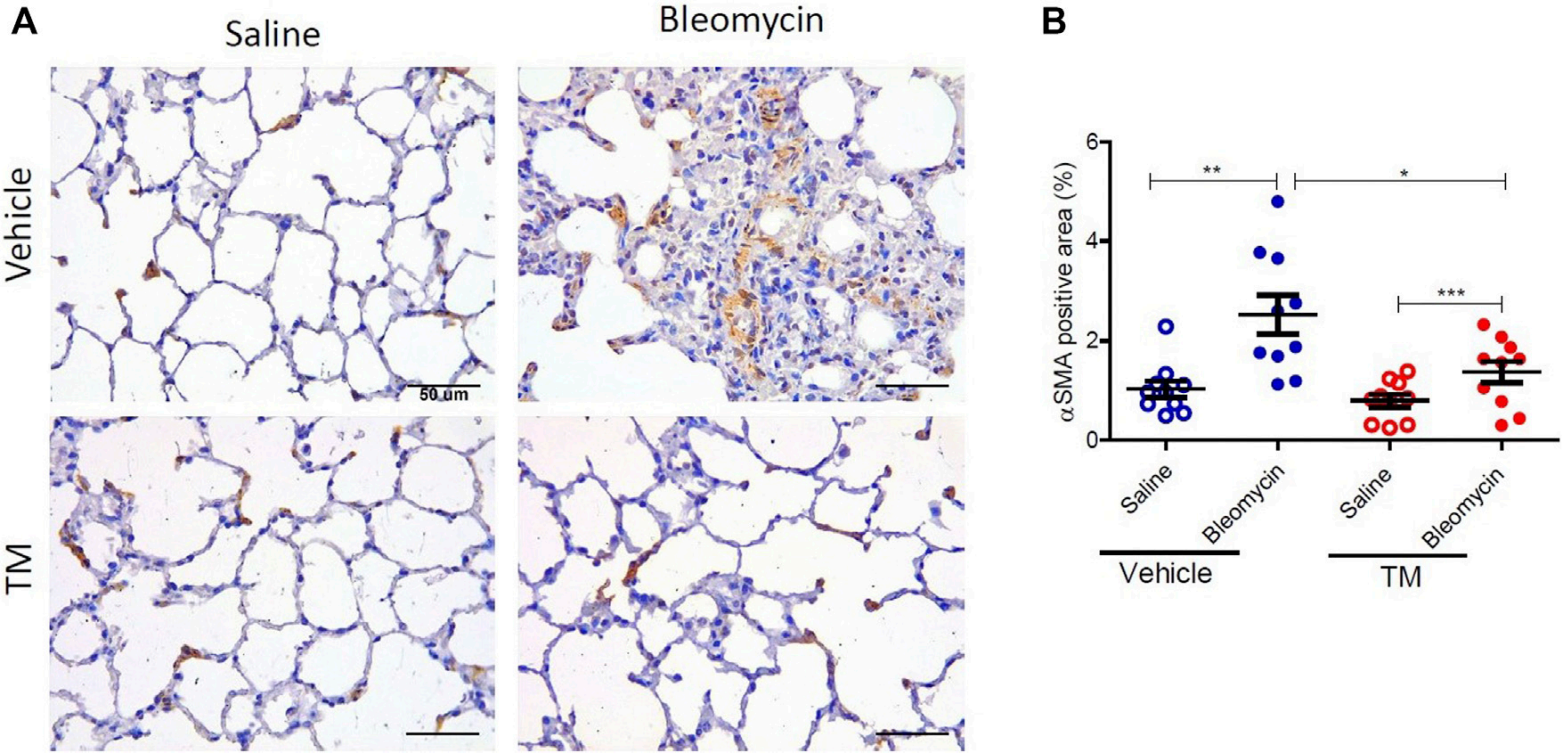
TM treatment significantly reduces bleomycin-induced activated myofibroblasts. **(A)** Representative photomicrographs of differentially treated lung tissue sections immuno-stained with an anti–αSMA antibody to identify contractile myofibroblasts. In bleomycin-infused segments, in the vehicle-control treated animals, more αSMA-positive cells were present in fibrotic remodeled areas compared to saline-infused segments of the same animals. In contrast, bleomycin-infused segments of sheep that were treated with TM had comparable staining with saline-infused segments. The αSMA-positive area was quantified in **(B)**. Significance was analyzed using a paired *t*-test between saline-*vs*. bleomycin-infused segments from the same animals and an unpaired *t*-test between segments from different animals (vehicle *vs*. TM treated animals), *n* = 10, *p < 0.05, **p < 0.01, ***p < 0.001. Scale bars = 50 µm.

To examine *in vivo* anti-fibrotic effects of TM treatment in our large animal model of bleomycin-induced pulmonary fibrosis, we assessed histopathology and collagen deposition in the parenchyma of differentially treated lung tissue segments as well as the expression of αSMA by immunohistochemistry (Figures 7–9). In vehicle-treated control sheep, bleomycin-infused lung segments had prominent fibrosis on histological sections, whereas saline-infused internal control lung segments in the same sheep had normal lung architecture with no signs of alveolar wall inflammation or fibrosis (Figure 7A). Lung injury and fibrosis were visibly reduced in lung segments that were infused with bleomycin in sheep treated with TM (Figure 7A). The overall pathology and collagen content was significantly reduced in bleomycin-infused segments of sheep treated with TM, compared to vehicle-treated control sheep (inflammation scores: bleomycin-vehicle 5.590 ± 0.4333 *vs*. bleomycin-TM 3.260 ± 0.5123, *p* = 0.0051; fibrosis scores: bleomycin-vehicle 9.911 ± 0.6670 *vs*. bleomycin-TM 5.480 ± 0.9799, *p* = 0.003; overall pathology scores: bleomycin-vehicle 15.5 ± 1.03 *vs*. bleomycin-TM 8.7 ± 1.5, *p* = 0.0028; Masson’s trichrome staining, % connective tissue/total field area: bleomycin-vehicle 6.84 ± 0.7% *vs*. bleomycin-TM 2.91 ± 0.3%, *p* < 0.0001) (Figures 7B–D; Figure 8B).

In vehicle-control sheep, bleomycin induced an increase in the percentage of alveolar tissue area (tissue percentage: saline-vehicle 44.81 ± 3.154% *vs*. bleomycin-vehicle 58.94 ± 3.18%, *p* = 0.0218). In sheep treated with TM, alveolar tissue area parameter was comparatively significantly improved (tissue percentage: bleomycin-vehicle 58.94 ± 3.18% *vs*. bleomycin-TM 45 ± 2.12%, *p* = 0.0018) (Figure 7E).

The expression of αSMA was also assessed by immunohistochemistry. In vehicle only treated sheep there was weaker staining for αSMA in saline-infused segments, whereas bleomycin-infused segments displayed strong staining in fibrotic remodeling areas (Figure 9A). In vehicle-treated sheep, the morphometric assessment showed a significant difference in αSMA staining intensity between the bleomycin-infused segment and the saline-infused segments (1.03 ± 0.16% saline-vehicle *vs*. 2.52 ± 0.39% bleomycin-vehicle, *p* = 0.0014) (Figure 9B). In bleomycin-infused segments of TM-treated animals while there was weaker staining for αSMA which was comparable to saline-infused segments of the vehicle only treated animals (Figure 9A), the morphometric analysis indicated that the difference with saline-infused segments of TM-treated animals was significant (1.37 ± 0.22 bleomycin-TM *vs*. 0.8 ± 0.13% saline-TM, *p* = 0.0007) (Figure 9B). When bleomycin-injured segments were compared between vehicle- and TM-treated animals, there was significantly less αSMA expression in TM-treated animals than vehicle only treated animals (2.52 ± 0.39% bleomycin-vehicle *vs*. 1.37 ± 0.22% bleomycin-TM), *p* = 0.0183 (Figure 9B).

## Discussion

Dysregulated angiogenesis has been implicated in the development and progression of pulmonary fibrosis. In the current study, we demonstrated that lowering bioavailable serum copper with tetrathiomolybdate chelation attenuates angiogenesis and fibrosis in our large animal model of bleomycin-induced pulmonary fibrosis. Moreover, tetrathiomolybdate treatment attenuated histopathological lesions, and improved lung function in bleomycin-induced pulmonary fibrosis. Tetrathiomolybdate also suppressed the accumulation of inflammatory cells in BAL fluid 2 weeks after bleomycin injury.

In the current study, bleomycin infusion into sheep lung segments increased angiogenesis. Studies performed on lung tissues taken from IPF patients, as well as in a number of animal models, demonstrate augmented angiogenesis in fibrotic lungs suggesting a role for dysregulated angiogenesis in the pathogenesis of pulmonary fibrosis (Keane et al., 1999a; Keane et al., 1999b; Keane et al., 2001; Strieter et al., 2002; Ebina et al., 2004; Burdick et al., 2005; Derseh et al., 2019). Furthermore, increased pulmonary vascular volume strongly predicted mortality in IPF patients (Jacob et al., 2017) signifying the importance of vascular remodeling in IPF. It is well known that normal wound healing requires strict coordination of events including angiogenesis and fibrocyte recruitment. By contrast, these processes are dysregulated in pulmonary fibrosis resulting in uncontrolled fibroblast proliferation, exuberant ECM accumulation, and the development of vascular remodeling associated with neo-angiogenesis (Strieter et al., 2007).

One of the key findings of this study was that the bleomycin-induced increases in CD34 and Collagen type IV-positive capillaries in fibrotic lung areas were attenuated in animals treated with TM. Antibodies against collagen type IV stains larger and more mature blood vessels (Rensing et al., 2012; Soltani et al., 2012), whereas small and newly formed microvessels are known to express CD34 on the cell surface (Delia et al., 1993). On the other hand, it has been reported that newly formed vessels are not collagen type IV positive (Rensing et al., 2012). In the current study, tetrathiomolybdate attenuated both collagen type IV and CD34 stained blood vessels indicating it can reduce both neovascularization and existing more mature vascular network. It is well known that angiogenesis is copper-dependent because many of the cytokines involved in this process are reliant on copper (Brewer, 2001; Brewer, 2005a; Goodman et al., 2005; Gupte and Mumper, 2009). TM is known for its antiangiogenic activity in many kinds of cancer patients. Reduction of copper levels by TM to 50–70% of baseline level has been demonstrated to inhibit cytokine signaling while housekeeping cellular copper needs are met (Askari et al., 2004; Brewer et al., 2004). Interestingly, in the current study, the copper concentration in TM-treated animals after 6 weeks of TM treatment was decreased to 73% compared to before-treatment baseline values, yet even with this relatively mild copper deficiency, TM achieved attenuation of bleomycin-induced angiogenesis and fibrosis.

In a previous study, we used the sheep model to show that the K_Ca_3.1 ion channel blocker, senicapoc was more effective in attenuating the increased vascularity in fibrotic lungs when compared with the anti-fibrotic drug pirfenidone (Derseh et al., 2019). A comparison between the previous and current studies, reveals that TM treatment was 31.65% more effective in reducing the density of CD34 stained capillaries in fibrotic lungs when compared to the FDA-approved drug pirfenidone (Derseh et al., 2019).

We found that bleomycin infusion not only potentiates an angiogenic response but also induces VEGF, a pro-angiogenic cytokine, that can promote neovascularization and is also thought to play a wider role in facilitating fibrogenesis (Fehrenbach et al., 1999; Hamada et al., 2005). Importantly, TM treatment suppresses bleomycin-induced VEGF expression in the lung. VEGF is a key mediator of angiogenesis (Carmeliet and Collen, 2000) and has been widely reported to be overexpressed in fibrotic lungs especially in animal models (Hamada et al., 2005; Hetzel et al., 2005; Wan et al., 2013). In IPF, VEGF is typically produced in increased amounts by both endothelial cells and alveolar type II epithelial cells in relatively preserved areas of the lung (Ebina, 2017). In our previous studies, we found augmented expression of VEGF in alveolar epithelial cells, airway epithelial cells, and capillary endothelial cells in the bleomycin-infused sheep lung segments (Organ et al., 2015a; Derseh et al., 2019). The importance of VEGF to the pathogenesis of pulmonary fibrosis has been shown in animal models of pulmonary fibrosis. In a rat model of pulmonary fibrosis, inhibition of the VEGF/VEGFR pathway with the angiogenesis inhibitor endostatin has been demonstrated to ameliorate pulmonary fibrogenesis (Wan et al., 2013). Similarly, in our previous study, reduction of VEGF via blockade of the K_Ca_3.1 channel with the drug senicapoc attenuated microvascular remodeling and fibrosis, as well as inhibiting capillary endothelial cell proliferation and fibroblast proliferation (Organ et al., 2017; Derseh et al., 2019). VEGF and other angiogenic cytokines are known to be copper-dependent (Brewer, 2001; Brewer, 2005a; Goodman et al., 2005; Gupte and Mumper, 2009). TM has been shown to inhibit VEGF production via suppression of NF-κB (Pan et al., 2002; Pan et al., 2003), a master switch for transcription of many of inflammatory and angiogenic cytokines. Whether this pathway is involved in the TM inhibition of VEGF expression in the current study is still to be determined. Overall, the reduced vascularity associated with the lowering of the angiogenetic drive would have likely contributed to the improvement of pathology and lung compliance in this model.

As well as attenuating fibrosis and angiogenesis, TM also significantly reduced inflammatory cell accumulation in BAL fluid 2 weeks after bleomycin injury, suggesting that TM can exert anti-inflammatory effects in addition to its anti-fibrotic and anti-angiogenic effects. This is consistent with previous reports in other animal models (Askari et al., 2004; Brewer et al., 2004; Ovet and Oztay, 2014). The mechanisms leading to lung fibrosis involve an intricate network of inflammatory cytokines and a variety of chemokines favoring the recruitment of inflammatory cells and fibroblasts, which ultimately leads to proliferation of fibroblasts, differentiation of myofibroblasts, followed by ECM deposition, and aberrant neovascularization (Agostini and Gurrieri, 2006). In our large animal model, infiltration of inflammatory cells is the initial response following bleomycin injury to the lung epithelium and vascular endothelium (Organ et al., 2015a). TM is known to inhibit a whole gamut of pro-inflammatory, pro-angiogenetic and pro-fibrotic cytokines underlying the pathogenesis of pulmonary fibrosis because these cytokines require higher levels of copper than is necessary for basic cellular needs (Brewer, 2003; Brewer et al., 2004; Brewer, 2005a; Brewer et al., 2006). While the complex mechanism(s) of how TM reduces fibrosis are yet to be fully elucidated, it remains a strong drug candidate as it influences multiple processes involved in pulmonary fibrosis.

The expression of alpha-smooth muscle actin (αSMA), an indicator of myofibroblast differentiation, is a key feature of active fibrosis. Myofibroblasts are key players in the pathogenesis and progression of pulmonary fibrosis. In the current study, αSMA expression was elevated by bleomycin injury in vehicle-treated animals, and this abnormal increase in αSMA expression was significantly reduced in TM-treated animals. Myofibroblasts are a primary source of collagen gene expression in fibrotic lesions in both IPF patients and animal models (Kuhn and McDonald, 1991; Zhang et al., 1994) These differentiated contractile fibroblasts have the capacity to produce excessive pro-inflammatory and pro-fibrogenic cytokines as well as ECM. In addition, myofibroblasts also produce angiogenic mediators and interact with endothelial cells modulating the angiogenic response (Bocca et al., 2015). Inhibition of the activation of myofibroblasts by TM may have contributed to its anti-fibrotic and anti-angiogenic effect. While the precise mechanism of how TM influences myofibroblast differentiation is not clear, it could involve downregulation of TGF-β1, as this cytokine is known to play a critical role in stimulating myofibroblast differentiation (Massagué, 2012). Support for this notion comes from a previous study reporting that TM reduces TGF-β1 expression in a bleomycin mouse model of pulmonary fibrosis (Brewer et al., 2004).

The strength of this study compared to other studies showing the protective/therapeutic effect of TM in pulmonary fibrosis models (Brewer et al., 2003; Brewer et al., 2004; Ovet and Oztay, 2014) is that we have been able to show that TM administration improved lung function concomitant with the attenuated pathology. In TM-treated animals, bleomycin-infused lung segments had improved lung compliance when compared with vehicle-treated sheep. Assessing physiological parameters is particularly important when undertaking clinical trials as physiologic improvements are usually included as clinical endpoints when assessing the efficacy of candidate drugs.

A limitation of the study was that the timing of the TM administration at 7 days after the final bleomycin infusion was a little earlier than our previous studies which administered antifibrotic drugs at 14 days after bleomycin. The comparatively shorter interval between bleomycin and drug treatment in the current study was undertaken to allow time for the TM therapy to reduce the bioavailable copper to therapeutic levels (Brewer et al., 2000; Brewer, 2003; Brewer et al., 2004; Pass et al., 2008). The timing of treatments in bleomycin models is important because the bleomycin-induced lung damage has an early inflammation phase followed by a later fibrotic phase (Jenkins et al., 2017). The early inflammation phase in bleomycin animal models largely subsides by day 7 after bleomycin. At this time-point, the later fibrotic phase establishes itself. The importance of these phases in terms of drug testing is that many anti-inflammatory treatments in bleomycin models are often typically only successful in ameliorating disease parameters when they are administered in the early phase of the model, and are not efficacious in the later fibrotic phase. In the current study, it is possible that the anti-inflammatory actions of TM could have a significant bearing on the study’s readouts. Indeed, Brewer et al. (2003) showed that increasing the delay time of TM administration after bleomycin, was associated with incrementally less fibrosis in terms of hydroxyproline content in the bleomycin-damaged mouse lung. Their study suggests that TM has stronger anti-inflammatory effects compared to its antifibrotic effects. In our study, due to the relatively early start of TM administration at the start of the fibrotic phase, we cannot rule out the possibility that the anti-inflammatory actions of TM may also make a significant contribution to improved readouts in this model of bleomycin-lung damage. What is clear is that TM does attenuate the bleomycin-induced increase in blood vessels in lung parenchyma and this parameter is likely to contribute to the improved lung function and pathology we observed with TM treatment.

In conclusion, our data demonstrate that TM is effective at attenuating both bleomycin-induced angiogenesis and pulmonary fibrosis. TM not only improved histopathology, but also improved lung function as measured by segmental lung compliance. Anti-angiogenic and anti-fibrotic effects of TM were most likely via the inhibition of pro-angiogenic and pro-fibrotic cytokines. The molecular mechanism(s) underpinning copper modulation of fibrotic pathways are an important area for future investigation, and this pathway represents a potential therapeutic target for pulmonary fibrosis.

## Data Availability

The raw data supporting the conclusions of this article will be made available by the authors, without undue reservation.
