# Supplementary material for: Tetrathiomolybdate Treatment Attenuates Bleomycin-Induced Angiogenesis and Lung Pathology in a Sheep Model of Pulmonary Fibrosis
Source: Front Pharmacol. 2021 Oct 22;12:700902. doi: 10.3389/fphar.2021.700902 (PMC8570673; doi:10.3389/fphar.2021.700902)
Supplement: Supplementary file 1 [file Image1.pdf]

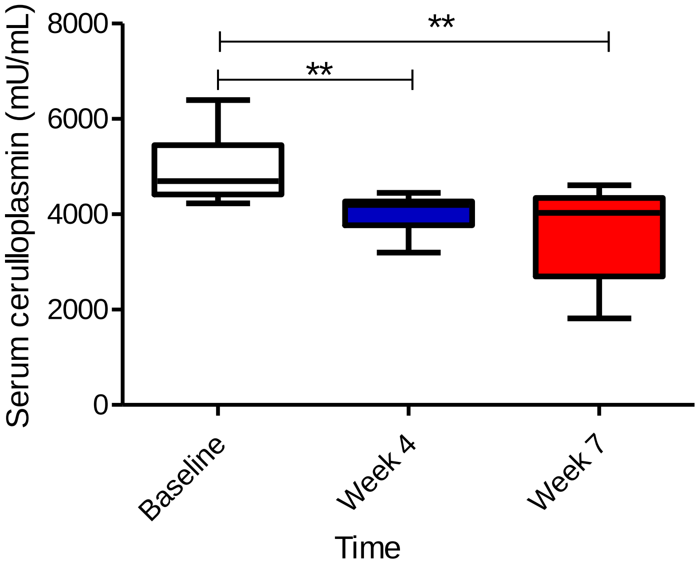

Supplementary Figure S1: **Serum ceruloplasmin concentration as an indication of copper levels at baseline, week four, and week seven following bleomycin injury.** TM treatment significantly reduced serum ceruloplasmin level, paired *t* test, $^{**}p < 0.01$ vs baseline.
